# Acute resistance to BET inhibitors remodels compensatory transcriptional programs via p300 co-activation

**DOI:** 10.1182/blood.2022019306

**Published:** 2025-02-13

**Authors:** Viral Shah, George Giotopoulos, Hikari Osaki, Markus Meyerhöfer, Eshwar Meduri, Aaron Gallego-Crespo, Malte A Behrendt, Maria Saura-Pañella, Aarti Tarkar, Benedict Schubert, Haiyang Yun, Sarah J Horton, Shuchi Agrawal-Singh, Patricia S Haehnel, Faisal Basheer, Dave Lugo, Ioanna Eleftheriadou, Olena Barbash, Arindam Dhar, Michael WM Kühn, Borhane Guezguez, Matthias Theobald, Thomas Kindler, Paolo Gallipoli, Paul Yeh, Mark A. Dawson, Rab K Prinjha, Brian JP Huntly, Daniel Sasca

**Affiliations:** 1 Department of Hematology and Oncology, University Medical Center of the Johannes Gutenberg University, Mainz, Germany; 2 University Cancer Center, University Medical Center of the Johannes Gutenberg University, Mainz, Germany; 3 German Cancer Consortium (DKTK), Frankfurt/Mainz, Germany; 4 Cambridge Stem Cell Institute, Cambridge, United Kingdom; 5 Department of Haematology, University of Cambridge, Cambridge, United Kingdom; 6 GlaxoSmithKline R&D, Collegeville, United States of America; 7 Department of Medicine V, Hematology, Oncology and Rheumatology, University of Heidelberg, Heidelberg, Germany; 8 Adaptive Immunity and Immuno-epigenetics Research Unit, GlaxoSmithKline R&D, Stevenage, United Kingdom; 9 Centre for Haemato-Oncology, Barts Cancer Institute, Queen Mary University of London, Charterhouse Square, London, United Kingdom; 10 Monash Haematology, Monash Health and School of Clinical Sciences at Monash Health, Monash University, Melbourne, Australia; 11 Department of Clinical Haematology, Peter MacCallum Cancer Centre, Melbourne, Australia

**Keywords:** Resistance evolution, AML, chromatin regulation, BET, p300

## Abstract

Initial clinical trials with drugs targeting epigenetic modulators - such as bromodomain and extraterminal protein (BET) inhibitors - demonstrate modest results in acute myeloid leukemia (AML). A major reason for this involves an increased transcriptional plasticity within AML, which allows cells to escape the therapeutic pressure. In this study, we investigated immediate epigenetic and transcriptional responses following BET inhibition and could demonstrate that BET inhibitor-mediated release of BRD4 from chromatin is accompanied by an acute compensatory feedback that attenuates down-regulation, or even increases expression, of specific transcriptional modules. This adaptation is marked at key AML maintenance genes and is mediated by p300, suggesting a rational therapeutic opportunity to improve outcomes by combining BET- and p300-inhibition. p300 activity is required during all steps of resistance adaptation, however, the specific transcriptional programs that p300 regulates to induce resistance to BET inhibition differ in part between AML subtypes. As a consequence, in some AMLs the requirement for p300 is highest during earlier stages of resistance to BET inhibition, where p300 regulates transitional transcriptional patterns that allow leukemia-homeostatic adjustments. In other AMLs, p300 shapes a linear resistance to BET inhibition and remains crucial throughout all stages of the evolution of resistance. Altogether, our study elucidates the mechanisms that underlie an “acute” state of resistance to BET inhibition, achieved through p300 activity, and how these mechanisms remodel to mediate “chronic” resistance. Importantly, our data also suggest that sequential treatment with BET- and p300-inhibition may prevent resistance development, thereby improving outcomes.

## Introduction

Dysregulated epigenetic and transcriptional processes are central determinants of Acute Myeloid Leukemia (AML) plasticity^2–5^. Consequently, modulators of aberrant transcriptional activation are, theoretically, attractive therapeutic targets, with BET inhibitors serving as a paradigm of novel epigenetic treatments in AML^6–8^. BET inhibitors predominantly target BRD4, an epigenetic reader that recognizes acetylated histones H3/H4 and transcription factors (TF), mediates transcriptional elongation/enhancer-directed transcription and serves for the recruitment of co-factors^9–12^. BRD4 commonly associates with cancer-maintenance genes (e.g. *MYC, RUNX1*) that are decorated with high-level acetylation, which leads to selective cancer dependency on BRD4^13^.

A major issue with BET inhibitors became apparent after their introduction into clinical trials. This consisted of modest response rates as monotherapy, due to either primary or acquired resistance^14–18^. As has been demonstrated for resistance to other novel epigenetic drugs, BET inhibitor resistance appears predominantly non-genetic, suggesting that epigenetic adaptation underlies this process^14–16,19–21^. This is further evidenced by the rapid restoration of *MYC* transcription in BET inhibitor-resistant cells despite continued BRD4 inhibition, or by recent reports about treatment strategies to target BET inhibitor-resistant cells, using epigenetic inhibitors to LSD1 or CDK7^14,16,22,23^.

AML usually respond to initial treatment but frequently later relapse. The development of resistance requires both the initial survival and immediate adaptation under the selective therapeutic pressure^24,25^. However, the mechanisms that underpin these acute processes are poorly understood. Furthermore, it remains unexplained as to whether mechanisms underpinning initial adaptation and later, full-blown resistance are uniform or further evolve. Obtaining such knowledge will allow better design of upfront treatments and possible maintenance strategies.

Here, we hypothesize that transcriptional plasticity in AML mediates early pathways of adaptation to, and escape from, BET inhibition. We predict that this plasticity provides new epigenetic vulnerabilities that, when properly inhibited, may extinguish later resistance.

## Methods

## Results

### Compensation for BRD4 exclusion from chromatin via BET inhibition occurs through acute redistribution of p300 to critical AML maintenance genes

To probe early responses across the genetic spectrum of AML, we treated 6 cell lines with the BET inhibitor iBET-151 (hereafter BETi)^6^ (Fig.S1A,Supp.Table_S1). We and others have extensively documented the effects of BET inhibition in KMT2A-rearranged or *NPM1*-mutated AML^6,8,13^. Complementary to previous data, we found that samples with *RUNX1-RUNX1T1*-rearrangement (KASUMI-1 and SKNO1 cell lines and two primary patient samples) exhibited the highest sensitivity to BETi, with significant reductions in viability and colony numbers (Fig.S1B-E). To validate the effects of BETi in *RUNX1-RUNX1T1-rearranged* AML *in-vivo*, we used an *Aml1-Eto9a*-driven murine AML model^27^. *Aml1-Eto9a-expressing* cKIT-high/Ter-119-negative murine fetal liver cells were either transplanted into mice or serially plated in methylcellulose (Fig.S1F). Transformation occurred in both settings, but BETi treatment led to a significant reduction in colony numbers *in-vitro* and extended disease latency/improved survival *in-vivo* (Fig.S1G-L).

Leukemia maintenance in *RUNX1-RUNX1T1*-rearranged AML relies on a refined balance between RUNX1-RUNX1T1 and wild-type RUNX1^28–32^. Cooperative binding of PU.1, FLI1, ERG and LMO2^30,33^ influences this balance, while p300/CREBBP mediates local activation through acetylation of histones/TFs^34–36^ and recruitment of BRD4 to facilitate transcriptional elongation at paused promoters^37,38^. As the mechanisms underlying *RUNX1-RUNX1T1*-rearranged AML maintenance are described in detail, this model was deemed optimal to deconvolute early chromatin processes upon BETi (overview in Fig.S2A).

**Figure 1:**
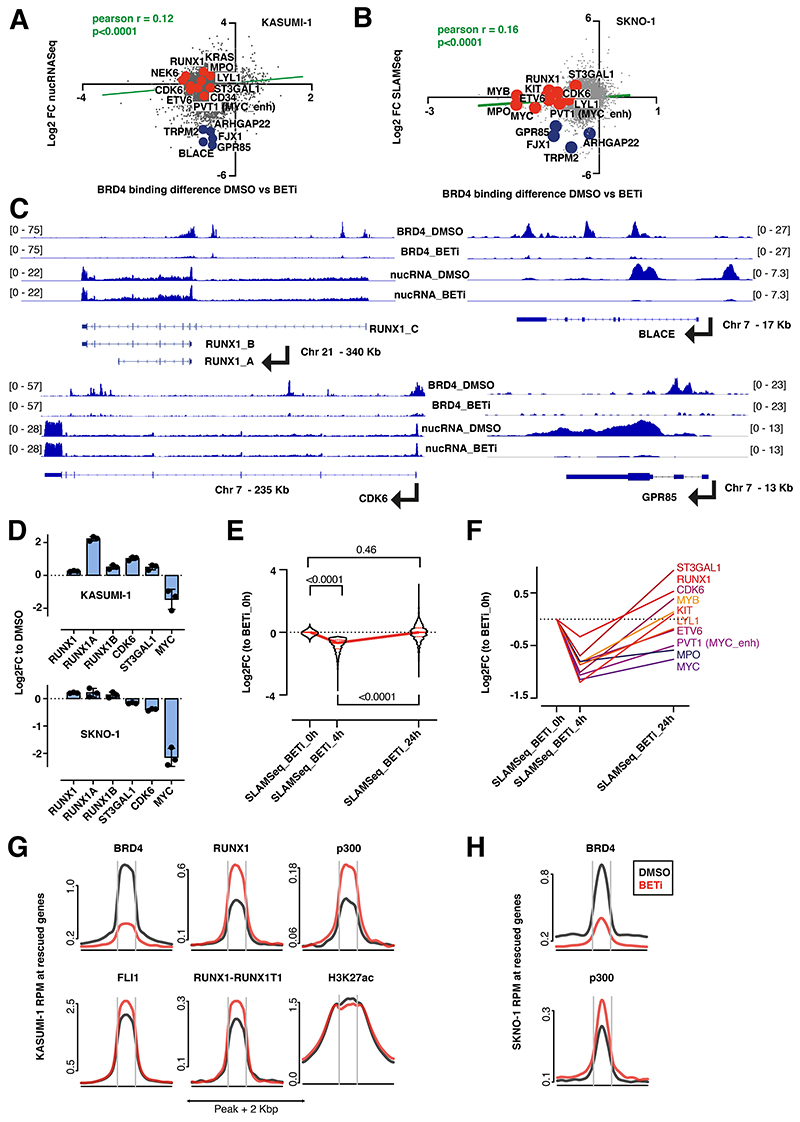
Compensation for the exclusion of BRD4 from chromatin via BET inhibition occurs through acute redistribution of p300 to critical AML maintenance genes A.Correlation of nucRNASeq with BRD4 binding dynamics in DMSO versus BETi-treated KASUMI-1 cells. Shown are signals that matched to both BRD4 and nucRNASeq peaks. Signals were annotated to the nearest promoters. B. Correlation of SLAMSeq with BRD4 binding dynamics in DMSO versus BETi-treated SKNO1 cells. BRD4 peaks were annotated to the nearest promoters and matched to the corresponding 3’UTR regions from SLAMSeq. C. Examples of BRD4 binding and nucRNASeq profiles in DMSO and BETi-treated KASUMI-1 cells, to demonstrate the rescue of mRNA production at/near promoters of the main RUNX1 isoforms and CDK6 upon BETi, but not at/near the BLACE or GPR85 promoters. D. Analysis of qPCR expression of the indicated transcripts/genes in KASUMI-1 and SKNO1 cells after treatment with either DMSO or BETi (24h). Shown are log2 Fold Changes normalized to DMSO-treated controls and SD from 3 biological replicates. E. SLAMSeq intensity at the 933 rescued genes at 0 hr (DMSO), 4 hr and 24 hr following BET inhibition. Shown are log2 fold change ratios compared to 0 hr (DMSO-treated controls). Three biological replicates were acquired per condition/time point. F. SLAMSeq intensity at the indicated exemplar rescued genes at 0 hr (DMSO), 4 hr and 24 hr following BET inhibition. Shown are log2 fold change ratios compared to 0 hr (DMSO-treated controls). Three biological replicates were acquired per condition/time point. G. Average binding curve profiles for BRD4, p300, RUNX1, FLI1, RUNX1-RUNX1T1 and H3K27ac in DMSO and BETi-treated KASUMI-1 cells, to demonstrate the BETi-triggered increase of p300 and RUNX1 binding at/near the rescued genes. H. Average binding curve profiles for BRD4 and p300 in DMSO and BETi-treated SKNO1 cells, to demonstrate the BETi-triggered increase of p300 binding at/near the rescued genes.

We assessed binding of BRD4 via chromatin immunoprecipitation followed by sequencing (ChIP-Seq) in KASUMI-1 and SKNO1 cells at 24hr post-treatment. This was long enough to allow release of BRD4 from chromatin, but prior to the onset of cell-cycle arrest/apoptosis (Fig.S2B-C). To link this with dynamics of transcription, we measured differential nuclearRNASeq (nucRNASeq) in KASUMI-1 and *de-novo* transcription via SLAMSeq in SKNO1 cells following 24hr of treatment (Fig.1A-B). As expected, BETi excluded BRD4 from chromatin (Fig.1A-C,2A-B). Surprisingly, at many essential AML maintenance genes, including *ETV6, MYC, CDK6, ST3GAL1, CD34* and the *MYC* enhancer *PVT1*, loss of BRD4 provoked only modest reductions in nuclear mRNA levels or *de-novo* transcription (Fig.1A-B). Furthermore, loss of BRD4 did not consistently lead to decreased transcription; for example, *RUNX1, LYL1, KRAS, MPO* and *NEK6* in KASUMI-1 and *RUNX1, MYB, KIT, CDK6* and *ST3GAL1* in SKNO1 showed increased transcription after 24hr of BETi (Fig.1A-C). Meanwhile, at non-essential transcripts such as *BLACE, GPR85, FJX1* or *TRPM2*, BRD4 exclusion significantly decreased transcription (Fig.1A-C). Due to this variance, global dynamics of transcription correlated only weakly with the degree of BRD4 exclusion (Fig.1A-B). We confirmed the blunting of BETi effects on *RUNX1* isoforms, *ST3GAL1*, and *CDK6* using qPCR, showing higher or only marginally decreased expression levels after 24hr of BETi (Fig.1D).

These data suggested that the transcriptional down-regulation of certain essential AML genes might have been protected through other epigenetic processes acutely after the loss of BRD4, thereby rescuing critical programs. To formally demonstrate this, we performed SLAM-Seq at baseline (0hr=DMSO-control) and compared it with SLAM-Seq after 4 and 24hr of BETi treatment in SKNO1. In a set of 933 genes, transcription significantly decreased at 4hr but recovered at 24hr of BETi, demonstrating that, after an initial impairment, these genes were indeed “rescued” at 24hr following treatment (Fig.1E). Representative rescued genes again included *ST3GAL1, RUNX1, CDK6, KIT, MYB, LYL1, ETV6, MPO, MYC* and *PVT1* (Fig.1F,S2D).

In addition to BRD4, we performed ChIP-Seq for central drivers and regulators of *RUNX1-RUNX1T1-*rearranged AML, including RUNX1, RUNX1T1(ETO), PU.1, FLI1, ERG, LMO2, p300, CDK9, the histone mark H3K27ac, and assessed chromatin accessibility dynamics via ATAC-Seq (Fig.S2A). We aligned these profiles with the regulatory regions of the 933 rescued genes in DMSO- vs. BETi-treated KASUMI-1 cells after 24hr of treatment. Contrary to the depletion of BRD4, p300 and RUNX1 binding increased markedly under BETi (Fig.1G). There was a moderate increase in FLI1, RUNX1T1 and ATAC-Seq signals, while H3K27ac and PU.1 levels remained unchanged (Fig.1G,S2E). LMO2 and CDK9 exhibited a moderate decrease and ERG a significant decrease in binding near the rescued genes (Fig.S2E). Of note, an increased p300 binding at the rescued genes, where BRD4 was significantly reduced, was also present in SKNO1 (Fig.1H). Taken altogether, these results demonstrate an unanticipated transcriptional compensation of critical genes following BETi, whereby p300 appears to play a central role.

### p300 compensates for the loss of BRD4 to rescue transcription after BET inhibition

**Figure 2:**
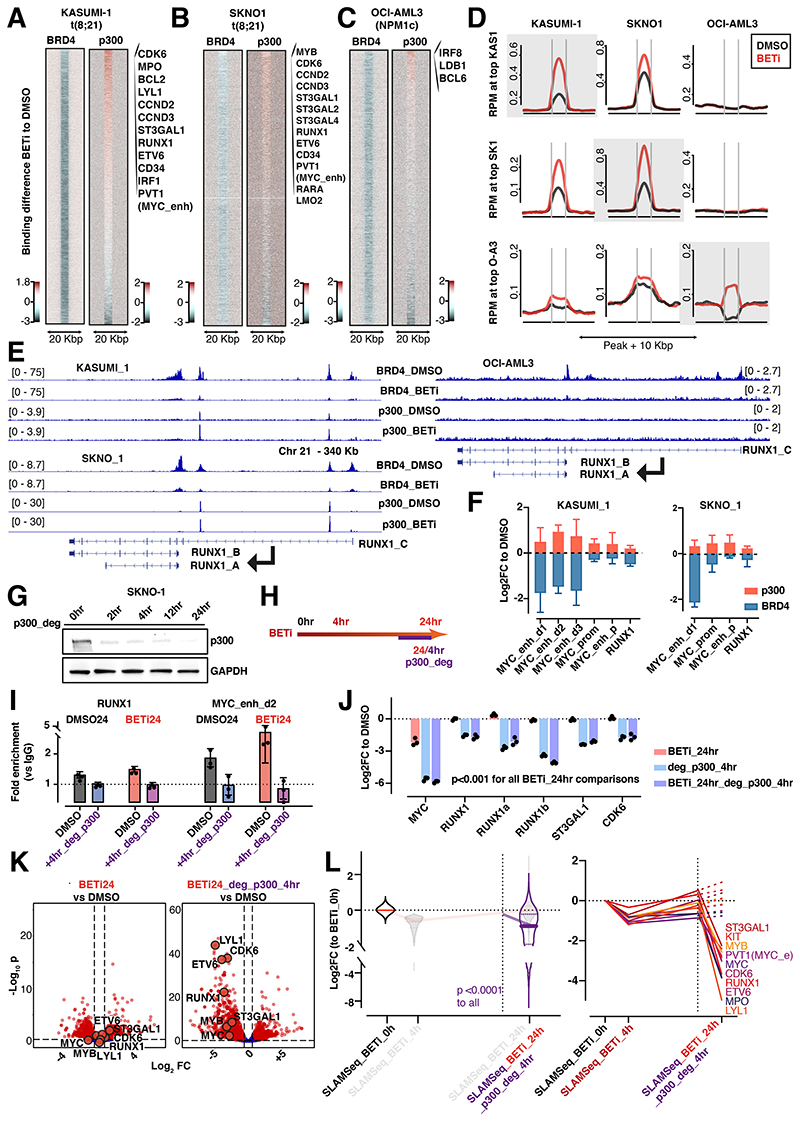
p300 compensates for the loss of BRD4 to rescue transcription after BET inhibition A.-C. Tornado plots of BRD4 and p300 binding differences between BETi and DMSO conditions in KASUMI-1, SKNO1 and OCI-AML3 cells (24h treatment). Positive enrichment (in red) shows stronger binding upon BETi. Negative enrichment, colored in blue, shows loss of binding upon BETi. Shown are results from representative matched pairs (color-tri: red = positive enrichment upon BETi treatment; white = no change; petrol blue = negative enrichment upon BETi treatment). With the exception of BRD4 ChIPSeq in KASUMI-1, which was performed once per condition, all experiments were performed with 2 biological replicates per condition. D. Average binding curve profiles for p300 at the top 5% rescued sites in the indicated cell lines. Upper panels show binding at the 5% best-scoring sites that were found in KASUMI1, middle panels show binding profiles at the 5% best-scoring sites that were found in SKNO1, lower panels apply the same method for 5% best-scoring sites that were found in OCI-AML3. E. Examples of BRD4 and p300 binding profiles in DMSO and BETi-treated KASUMI-1, SKNO1 and OCI-AML3 cells, to demonstrate the BETi-triggered increase of p300 binding at/near promoters of the main RUNX1 isoforms. F. Bar plots of BRD4 (blue bars) and p300 (red bars) log2 fold changes of binding between the BETi and DMSO conditions in KASUMI-1 and SKNO1 cells (24h treatment) at the indicated *MYC* and *RUNX1* regulatory regions. Shown are log2 fold changes of ChIP-qPCR results from 3 biological replicates and SD. G. Western blot for p300 and GAPDH in SKNO1 cells during a time lapse of 0 (DMSO-treated control), 2, 4, 12 and 24 hr of PROTAC-mediated p300 degradation via deg_p300. H. Scheme of treatment with BETi for 24 hr and degradation of p300 within the last 4 hr of BETi. I. Bar plots of p300 binding ratios to IgG in BETi and DMSO conditions with or without the addition of deg_p300 within the last 4 hr of treatment in SKNO1 cells. Shown are ChIP-qPCR results from 3 biological replicates at the *RUNX1* promoter and one *MYC* enhancer and SD. J. Analysis of qPCR expression of the indicated transcripts/genes in SKNO1 cells after treatment with either BETi (24h of treatment), deg_p300 (4h) or BETi (24h) with the addition of deg_p300 during the last 4h of treatment. Shown are log2 Fold Changes normalized to DMSO-treated controls and SD from 3 biological replicates. K. Volcano plots showing SLAMSeq expression changes in SKNO1 cells after 24 hr of treatment with DMSO or BETi (same as Fig.S1E) and DMSO or BETi plus the addition of deg_p300 within the last 4h of treatment. Three biological replicates were acquired per condition/time point. L. (Left panel) SLAMSeq intensity at the 933 rescued genes at 24 hr of BET inhibition with the addition of deg_p300 during the last 4 hr of treatment, to be compared with the values at 0 hr (DMSO-treated controls), 4 hr and 24 hr of BETi treatment in the background. Shown are log2 fold change ratios to 0 hr (DMSO-treated controls). (Right panel) SLAMSeq intensity at the indicated exemplar rescued genes at 24 hr of BET inhibition with the addition of deg_p300 during the last 4 hr of treatment, to be compared with the values at 0 hr (DMSO), 4 hr and 24 hr of BETi treatment in the background. Shown are log2 fold change ratios to 0 hr (DMSO). Three biological replicates were acquired per condition and time point.

To confirm whether p300 was preferentially redistributed at/around rescued genes, we integrated the dynamics of BRD4 binding with those of other TFs/regulators at a global scale. In both KASUMI-1 and SKNO1, p300 binding preferentially increased at sites corresponding to critical AML maintenance genes such as *CDK6, CCND2-3, ST3GAL1-2*,*4, RUNX1, ETV6, CD34* and *PVT1*, which also coincided with many of the exemplar rescued genes (Fig.2A-B,E,S3C). To observe whether such a locus-specific accumulation of p300 after BETi was reproducible in other AML subtypes, we performed ChIP-Seq for BRD4 and p300 (again at 24hr following DMSO/BETi) in a completely independent BETi-sensitive AML cell line—OCI-AML3 (*NPM1*-mutated). Here, we did observe an increase of p300 at distinct loci, but these were fewer and exemplified by a different set of genes, including *IRF8, LDB1* and *BCL6* (Fig.2C,S2D).

We next compared signal intensities across all three models for the top 5% of sites with increased p300 binding in each cell line. Upon BETi, p300 signals that were in the top 5% in KASUMI-1 were also significantly increased in SKNO1, and vice versa (Fig.2D). In contrast, OCI-AML3 displayed a distinct pattern, characterized by generally lower p300 binding to chromatin and minimal overlap with the top 5% sites from KASUMI-1/SKNO1 (Fig.2D,S2D). Comparing differential binding of the regulators presented above, either globally or solely at the top 5% of KASUMI-1 p300-increased sites, we observed significant enrichment for RUNX1, RUNX1T1 and FLI1 and a marginally higher intensity for H3K27ac (Fig.S3A-B). All other parameters demonstrated either no change or a decrease in binding upon BETi (Fig.S3A or not shown). Reciprocal binding of BRD4 and p300 upon BETi was further confirmed through quantitative ChIP-qPCR in KASUMI-1 and SKNO1 cells at the *MYC* promoter, three *MYC* enhancers and the *RUNX1* promoter (Fig.2F).

To confirm the role of p300 in transcriptional compensation at rescued genes functionally, we used a PROTAC-based p300 degrader (p300_deg)^39^. Time-resolved western blots of p300 expression, along with matched qPCR for *MYC, RUNX1a* and *RUNX1b*, confirmed a significant loss of p300 expression and its targets within 4hr of p300_deg (Fig.2G,S4A-D). This loss persisted at similar levels after 12- and 24hr but was not accompanied by any phenotype change or increased apoptosis within this timeframe (Fig.2G,S4C-D). In line with the experiments leading to the discovery of the rescued genes, we treated SKNO1 and KASUMI-1 with BETi or DMSO for 24hr, also adding either p300_deg or a mock control during the last 4hr (Fig.2H). ChIP-qPCR at *RUNX1* and one *MYC* enhancer showed that p300 binding was depleted upon p300_deg (Fig.2I). qPCR of the rescued genes *MYC, RUNX1 (+RUNX1a* and *RUNX1b), ST3GAL1* and *CDK6* showed a dramatic loss of expression upon p300_deg in both DMSO- and BETi-pretreated SKNO1 and KASUMI-1 cells (Fig.2J,S4E). Finally, we performed SLAMSeq at 24hr in BETi-treated SKNO1 cells with the addition of either a mock control or p300_deg for the last 4hr. *De-novo* transcription of rescued genes, which was blunted or increased in BETi-treated cells, decreased dramatically upon p300_deg (Fig.2K-L). Once again, this was most evident for *ST3GAL1, KIT, MYB, PVT1, MYC, CDK6, RUNX1, ETV6 MPO* and *LYL1* (Fig.2K-L). Altogether, our findings demonstrate that p300 is critically involved in the acute rescue of important maintenance genes of *RUNX1-RUNX1T1*-rearranged AML.

### Sequential pharmacological BET-followed by p300-inhibition suppresses expression of rescued programs

**Figure 3:**
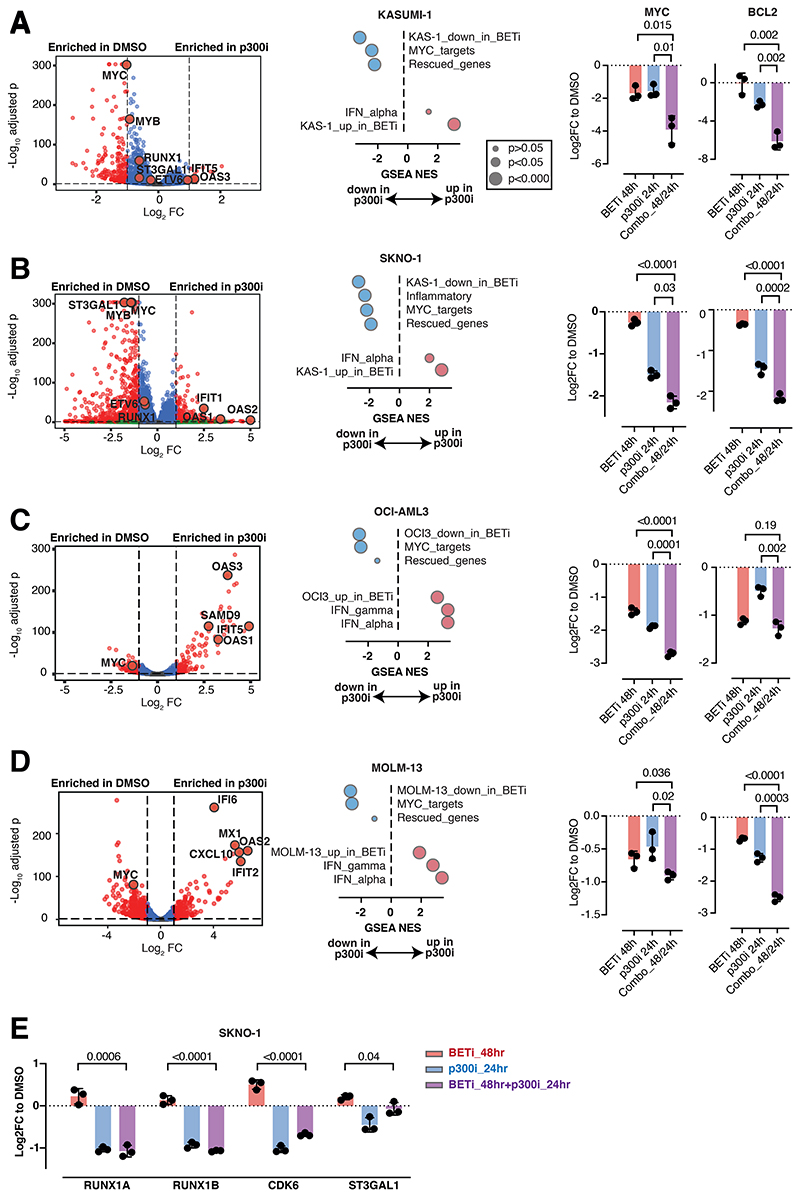
Sequential pharmacological BET-followed by p300-inhibition counteracts expression of rescued programs A.-D. Left panels - Volcano plots showing RNASeq expression changes in the indicated cell lines after 24h of treatment with DMSO or p300i. Middle panels - Dot plots of GSEA normalized enrichment scores (NES) and FDRq significance of the top-scoring data sets after treatment with either DMSO or p300i for 24h in the indicated cell lines. Right panels - Analysis of qPCR expression of MYC and BCL2 in the indicated AML cell lines after treatment with either DMSO (48h of treatment), BETi (48h), p300i (24h) or sequential BETi and p300i (48h/24h). Shown are log2 Fold Changes normalized to DMSO-treated controls and SD from 3 biological replicates. E. Analysis of qPCR expression of the indicated transcripts/genes in SKNO1 cells after treatment with either DMSO (48h of treatment), BETi (48h), p300i (24h) or sequential BETi and p300i (48h/24h). Shown are log2 Fold Changes normalized to DMSO-treated controls and SD from 3 biological replicates.

Given the overlap of critical AML maintenance genes with those rescued by p300 during acute adaptation to BETi, we hypothesized that these BETi-dependent transcripts are also regulated by p300. To test this, we performed RNASeq on KASUMI-1, SKNO1 as well as OCI-AML3 and MOLM-13 (a KMT2A-rearranged, BETi-sensitive cell line with transcriptional similarities to OCI-AML3) cells after 24hr of treatment with DMSO or the p300+CREBBP acetyltransferase-selective inhibitor A-485 (p300i). Significant decreases in several p300-rescued genes, including *RUNX1, ST3GAL1, ETV6, MYB*, and *MYC* were observed in KASUMI-1 and SKNO1 (Fig.3A-B), while *MYC* expression consistently decreased and standard interferon-stimulated genes (ISGs) increased in OCI-AML3 and MOLM-13 (Fig.3C-D). Aiming to record co-dependencies of p300i and BETi, we generated datasets of differentially up-/downregulated genes for each KASUMI-1, MOLM-13 and OCI-AML3^13^ cells treated with DMSO/BETi and from p300-rescued genes identified in SKNO1 via SLAM-Seq, and integrated these into the MSigDB Hallmark collection (Supp.Table_S3). Assessing gene set enrichment, we saw that all BETi-regulated gene sets were significantly enriched in their p300i-treated counterparts, with a decrease in MYC targets and an increase in ISGs observed across all models (Fig.3A-D). Importantly, rescued genes were enriched in SKNO-1 and KASUMI-1 but not in OCI-AML3 and MOLM-13, indicating AML subtype-specific p300-mediated compensation (Fig.3A-D).

Our results consistently highlighted MYC and its transcriptional program as key targets of both BETi and p300i across all datasets. We hypothesized that a redundant role for BET and p300 in the maintenance of critical AML genes might define the acute resistance and predict for p300i sensitivity. We determined the effects of sequential BETi followed by p300i on the transcription of *MYC* and the MYC-dependent gene *BCL2* across all 4 models. Single agent treatment with BETi or p300i strongly downregulated both genes in all cell lines. Sequential treatment, however, resulted in even greater downregulation of MYC in all models and BCL2 in all but the OCI-AML3 model (Fig.3A-D). We also conducted a MYC reporter assay in the unrelated HCT116 cell line, where combined treatment more effectively suppressed MYC activity than single-agent use (Fig.S5A). Finally, we determined the effects of sequential BETi followed by p300i on the transcription of the rescued genes *RUNX1_A, RUNX1_B, ETV6* and *ST3GAL1* in SKNO-1 and KASUMI-1. Whereas BETi triggered upregulation, single p300i and combined sequential treatment caused downregulation of these transcripts (Fig.3E,S5B).

These results demonstrate the dependency of genes that can rescue the apoptotic phenotype following BETi upon p300i, and also a putative clinical utility of sequential BET-followed by p300-inhibition to impair rescue programs.

### Sequential pharmacological BET-followed by p300-inhibition synergistically suppresses AML proliferation

**Figure 4:**
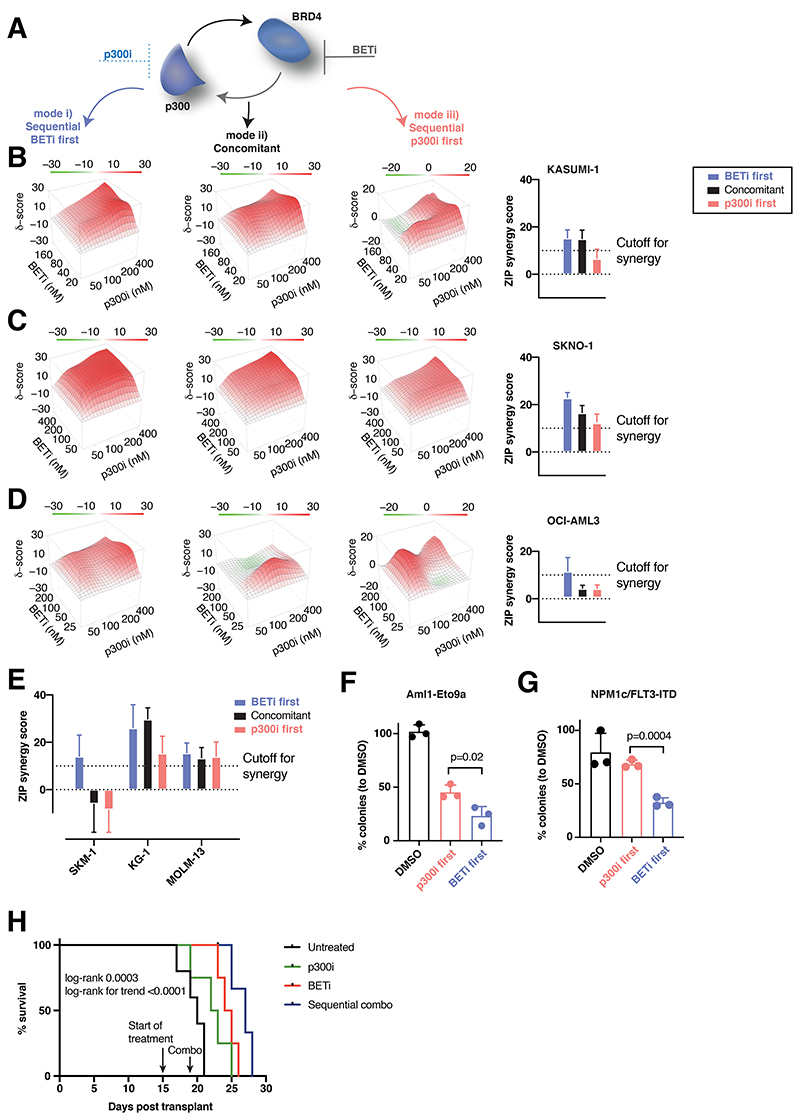
Sequential pharmacological BET-followed by p300-inhibition synergistically/optimally suppresses AML proliferation A. Schema of the approach adopted to inhibit the proposed feedback rescue loop to p300 after BETi. In total, 6 cells lines were treated, each in 3 biological replicates with the two inhibitors at each 4 dosages and DMSO (20 combinations) in 3 temporal sequences: mode i) BETi sequentially followed by p300i, mode ii) both inhibitors concomitantly and mode iii) p300i sequentially followed by BETi. B.-D. Three-dimensional diffusion plots indicating maximal synergy scores (right panels) of combined treatment with BETi and p300i in the indicated orders and cell lines. Treatment efficiency was measured with CellTiterGlo^**®**^ at day 5 after begin of the first treatment. For the sequential treatment modes, the second compound was added 48hr after initial treatment commencement. Shown are averages and (for bar plots) SD from 3 biological/experimental replicates. ZIP scores > +10 were considered synergistic, while ZIP scores < -10 were antagonistic. E. Plots of synergy scores of combined treatment with BETi and p300i in the indicated orders and cell lines. Treatment efficiency was measured with CellTiterGlo^**®**^ at day 5 after the beginning of the first treatment. For the sequential treatment modes, the second compound was added 48hr after treatment began. Shown are averages and SD from 3 biological/experimental replicates. F. Analysis of colony formation of Aml1-Eto9a-transformed murine AML cells after 2 rounds of plating and treatment with either DMSO in both plates, BETi followed by p300i or vice versa. Shown are mean percentages normalized to DMSO and SD from 3 biological replicates. In each plating, treatment was performed for 7 days in methylcellulose. G. Analysis of colony formation of Npm1c/Flt3-ITD murine AML cells after 2 rounds of plating and treatment with either DMSO in both plates, BETi followed by p300i or vice versa. Shown are mean percentages normalized to DMSO and SD from 3 biological replicates. In each plating, treatment was performed for 7 days in methylcellulose. H. Tertiary Aml1-Eto9a-transplanted nod-scid gamma (NSG) mice were allocated to 4 treatment groups (untreated (n=5), BETi-treated (n=4), p300i-treated (n=4) and sequentially combined treated (n=4)). Shown is the Kaplan-Meier plot of survival with log-rank p values (Mantel-Cox and trend). One mouse in the sequential combined treatment group was censored due to death to AML-unrelated reasons at d23.

Our data suggested an immediate compensatory mechanism to maintain transcription of critical genes via increased p300-dependency following BETi (Fig.4A). To determine if this response provided a therapeutic vulnerability, we treated KASUMI-1, SKNO1 and OCI-AML3 with the two inhibitors in 3 temporal sequences: mode i) BETi sequentially followed by p300i, mode ii) both inhibitors concomitantly and mode iii) p300i followed by BETi. Treatment was performed using BETi and p300i each at 4 dosages+DMSO-controls (20 combinations). This strategy allowed for an unbiased analysis of treatment effects and the determination of synergistic activity, as quantified using the ZIP algorithm^42^. Importantly, BETi+p300i treatment was invariably synergistic only in mode i) BETi-first (Fig.4B-D). KASUMI-1 and SKNO1, but not OCI-AML3, were synergistic in mode ii) (concomitant), while only SKNO1 cells remained synergistic in the p300i-first sequence (mode iii)) (Fig.4B-D).

To assess the effects of BETi+p300i across other AML genotypes, we extended the analysis to more BETi-sensitive cells—SKM1, MOLM-13, KG-1. Again, synergistic inhibition of proliferation was present in all cell lines in mode i) the BETi first – p300i second treatment sequence (Fig.4E,S6A). Inhibition became less synergistic using concomitant treatment in SKM1, while only KG-1 and MOLM-13 demonstrated synergism when p300i was used ahead of BETi (Fig.4E,S6A).

We also assessed for synergism in our models of *Aml1-Eto9a* and *Npm1*^flox-cA/+^;*Flt3*^ITD/+^;Mx1-Cre+(*Npm1c/Flt3-ITD*)^4,43^ murine AML, analyzing clonogenic capacity. Cells were plated in methylcellulose after BETi followed by p300i or vice-versa. While both temporal sequences demonstrated reduced colony numbers vs. DMSO, BETi followed by p300i was significantly more effective than the reverse sequence (Fig.4F-G). Finally, we performed an *in-vivo* study using our *Aml1-Eto9a* system. Tertiary transplanted mice were treated in four arms: vehicle compound, single BETi, single p300i and a sequential combo with BETi and p300i. Mice receiving the sequential combination showed extended survival compared to those in other treatment groups (Fig.4H), without an increase in toxicity to normal hematopoiesis (Fig.S6B-E). In summary, these results demonstrate increased therapeutic efficacy when BETi and p300i are combined. Consistent with our proposed p300-mediated mechanism for the acute adaptation of BETi-treated cells, sequential treatment starting with BETi is most efficient.

### p300i is invariably therapeutically effective during early stages of resistance to BET inhibition

**Figure 5:**
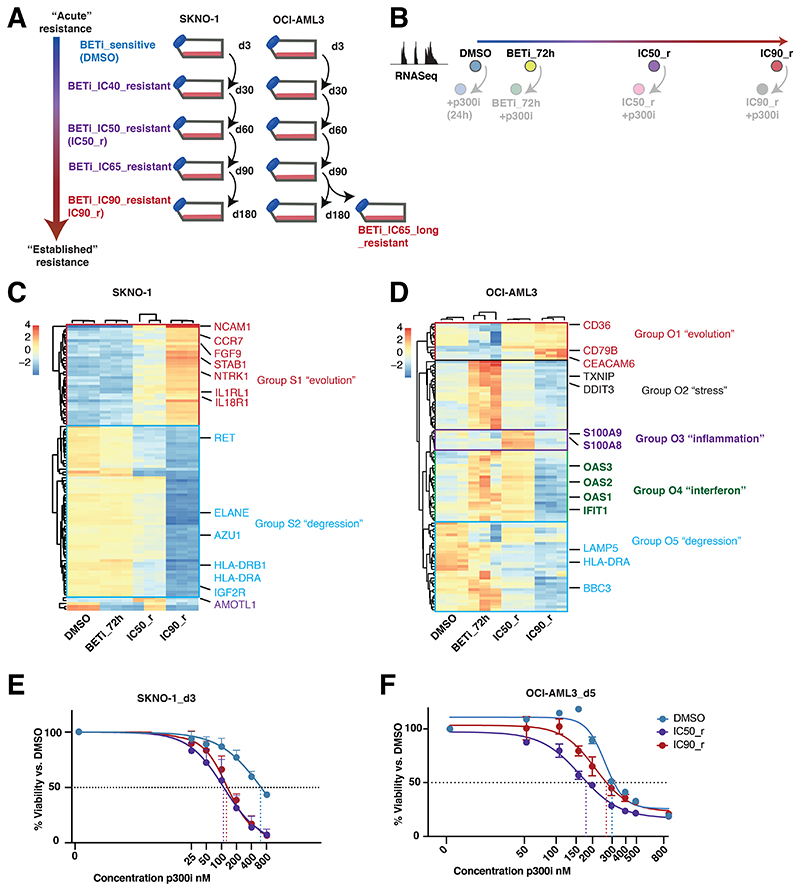
p300i is invariably effective during early stages of resistance to BET inhibition A. Graphical schema of the experimental approach to induce and store cells at all stages of resistance to BETi in SKNO1 and OCI-AML3 cells. B. Experimental approach to the assessment of transcriptional changes via RNASeq during the longitudinal scale of establishment of resistance to BETi. To avoid putative sequencing-related batch effects, these experiments were directly performed in conjunction with matched p300i-treated (24h) samples (shown with lower opacity). All RNASeq experiments were performed on 3 biological replicates that were raised from individual wells (altogether 24 RNASeq samples per model). C.-D. Variance by non-supervised hierarchical clustering of the 100 most variable genes in DMSO, BETi_72h, IC50_r and IC90_r isogenic SKNO1 (5C) and OCI-AML3 (5D) cells. E.-F. Assessment of cell proliferation of the indicated isogenic SKNO1 (5E) and OCI-AML3 (5F) cell lines after 72h (for SKNO1) and 120h (for OCI-AML3) of p300i treatment. Shown are mean percentages normalized to DMSO-treated controls and SD from 3 biological replicates.

Disease relapse in AML usually appears after months/years and whether the mechanisms underlying acute resistance persist at later timepoints is unknown. We simulated the evolution of resistance-acquisition to BETi in the two AML models that we had extensively tested so far: SKNO1 (*RUNX1-RUNX1T1*-rearranged) and OCI-AML3 (*NPM1*mut). We treated SKNO1 and OCI-AML3 cells with DMSO or increasing dosages of BETi at IC20 (SKNO1-only), IC40, IC50, IC65, IC80 (SKNO1-only) and IC90 concentrations to generate cell lines along a continuum from early to established resistance (Fig.5A,S7A-B).

We then assessed transcriptional changes during the evolution of resistance by performing RNASeq at 4 longitudinal time-points: DMSO-treated, short-term BETi-treated (BETi_72h), incipient resistance (IC50_r) and full-blown resistance to BETi (IC90_r) (Fig.5B). Non-supervised clustering of the most variable genes revealed two distinct transcriptional patterns in SKNO1, which we termed the “degression” module (Group_S2) for genes decreasing in expression, and the “evolution” module (Group_S1) for those increasing (Fig.5C). The evolution module was enriched for signaling ligands and receptors such as *NCAM1, CCR7, FGF9*, and *IL1RL1* (Fig.5C,S8C). In contrast, OCI-AML3, displayed a more complex and certainly non-linear transcriptional evolution of resistance, with 5 distinct modules. Besides a small “evolution” signature (Group_O1) and a larger “degression” group (Group_O5), some genes only increased transiently at lower IC doses of BETi (“stress” genes - Group_O2) (Fig.5D). Of importance, a small number of “inflammation”-associated genes (Group_O3) were transiently expressed only in incipiently resistant IC50_r cells and consisted of potential resistance modulators such as S100A9/S100A8^44–46^ (Fig.5D,S8E). Finally, we identified an “interferon” group (O4), which showed increased expression immediately after BETi treatment and in IC50_r but decreased in IC90_r cells (Fig.5D,S8G).

We next treated cells from all stages of BETi-resistance with p300i and measured proliferation. In SKNO1, BETi-resistance severely sensitized cells to p300i throughout all stages (Fig.5E,S7C-D). Sensitivity to p300i was highest starting with IC50_r and remained high during later stages of established resistance, including in IC90_r cells (Fig.5E,S7C-D). In OCI-AML3, p300i abrogated proliferation at lower IC50 values during all steps of resistance to BETi (Fig.5F,S7E-F). However, compared with stages of established resistance (IC65_long_r and IC90_r), cells at early-resistant stages required significantly less p300i to achieve the same inhibitory effects (Fig.5F,S7E-F).

Altogether, these results indicate complex cell-type/genotype-dependent transcriptional resistance mechanisms, but also uncover a broad, early genotype-independent window to enhance BETi responses with p300i.

### p300 regulates multiple downstream mediators of BET-inhibitor resistance

**Figure 6:**
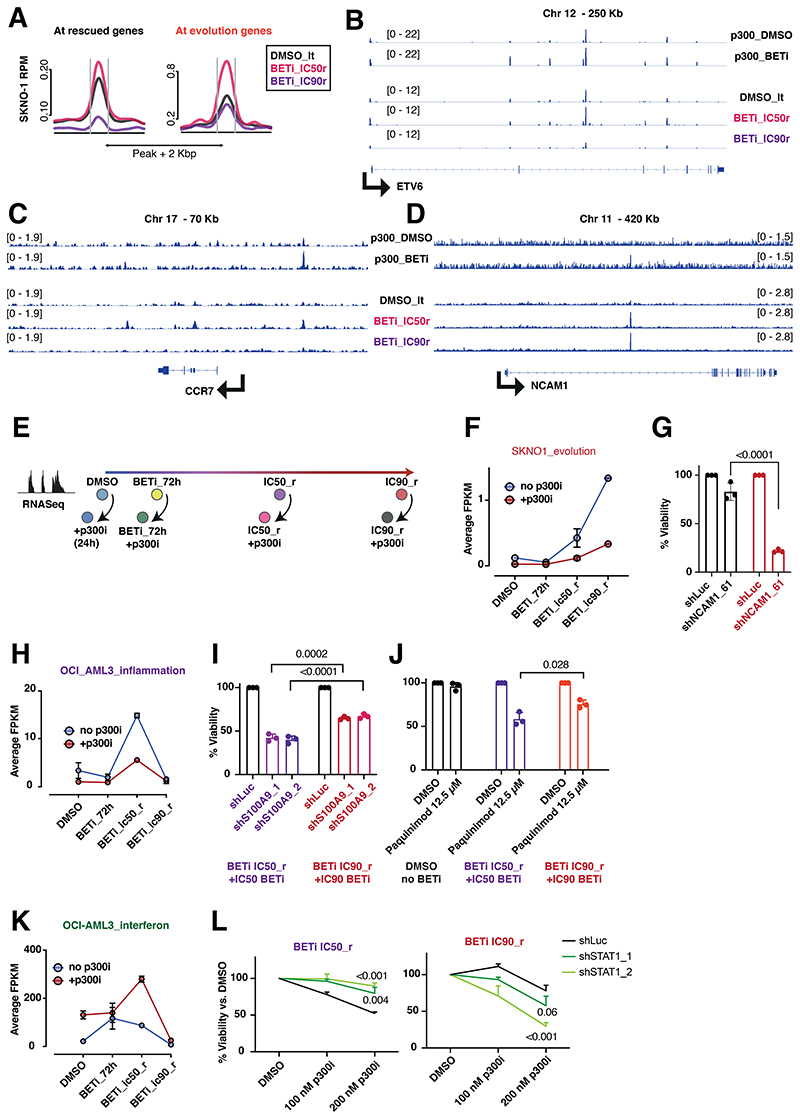
p300 regulates multiple downstream mediators of BET-inhibitor resistance A. Average binding curve profiles for p300 in the indicated isogenic SKNO1 with different degrees of BETi-resistance, centered at/around regulatory regions of the rescued (left panel) and evolution (right panel) genes. B.-D. Examples of binding kinetics of p300 at *ETV6* (a rescued gene) (B), *CCR7* (an evolution gene) (C) and *NCAM1* (an evolution gene) (D) in BETi-sensitive and BETi-resistant settings. E. Continued from Figure 5B - Experimental approach to the assessment of transcriptional changes during the longitudinal scale of establishment of resistance to BETi and sensitivity towards p300i. F. Longitudinal analysis of expression of SKNO1 evolution genes that code for ligands and signaling receptors, during all stages of resistance to BETi, with or without addition of p300i for 24h. Individual genes within the group were chosen based on their structural assignment per gene ontology analysis. Shown are average FPKM values from 3 biological replicates and SD. G. Assessment of cellular viability after *NCAM1* knockdown in the indicated SKNO1 isogenic cells for 120h. Shown are percentages normalized to Luc controls and SD from 3 biological replicates. H. Longitudinal analysis of expression of all OCI-AML3-related “inflammation” genes, during all stages of resistance to BETi, with or without addition of p300i for 24h. Shown are average FPKM values from 3 biological replicates and SD. I. Assessment of cellular viability after S100A9 knockdown in the indicated isogenic cells for 120h. Shown are percentages normalized to Luc controls and SD from 3 biological replicates. J. Analysis of cellular viability after treatment with either DMSO or Paquinimod in the indicated OCI-AML3 isogenic cells for 72h. Shown are percentages normalized to DMSO-treated controls and SD from 3 biological replicates. K. Longitudinal analysis of expression of all OCI-AML3-related “interferon” genes, during all stages of resistance to BETi, with or without addition of p300i for 24h. Shown are average FPKM values from 3 biological replicates and SD. L. Analysis of cellular viability after STAT1 knockdown and DMSO- or p300i-treatment for 120h in the indicated isogenic cell lines. Shown are percentages normalized to DMSO and SD from 3 biological replicates.

To assess p300 chromatin-binding dynamics during later stages of resistance to BETi, we performed ChIP-Seq for p300 in DMSO-treated, BETi_IC50_r, and BETi_IC90_r SKNO1. At rescued genes, p300 binding was significantly increased in IC50_r but nearly absent in IC90_r cells, indicating a possible shift to other epigenetic regulators or diminishing gene relevance with full resistance (Fig.6A-B). Similarly, at the Group_S1 genes, associated with resistance evolution in SKNO1, p300 binding was significantly increased in IC50_r cells and returned to levels similar to those in DMSO, a finding that appeared counterintuitive given the continued sensitivity of both IC50_ and IC90_resistant cells to p300i (Fig.6A,C-D). However, p300 binding increased significantly at precisely 3 sites in IC90_r cells compared to DMSO. The most notable/significant site was the promoter region of *NCAM1*, where p300 was also enriched in IC50_r (Fig.6D). Moreover, when comparing the initial 24hr timepoint between DMSO- and BETi, p300 had already begun to bind at the same regulatory region of *NCAM1* acutely following BETi.

We also assessed gene expression alterations following p300-inhibition at key stages of adaptation to BETi (DMSO, 72h_BETi, IC50_r, IC90_r) (Fig.6E). In SKNO1, p300i severely repressed many “evolution” genes in both IC50_r and IC90_r cells (Fig.6F,S8A,S8C). The top-scoring “evolution” gene, and one that was also significantly re-repressed by p300i, was *NCAM1* (Fig.5C,6D,6F). We previously demonstrated that *NCAM1* leads to resistance in diverse types of AML^47^. Furthermore, *NCAM1* associates with higher relapse rates in *RUNX1-RUNX1T1*-rearranged AML^48,49^. To demonstrate a direct role for NCAM1 in the development of resistance to BETi in SKNO1, we performed RNAi-mediated knockdown in DMSO- vs IC90_r cells. *NCAM1* knockdown severely abrogated cell growth of IC90_r cells, while barely repressing proliferation of DMSO cells (Fig.6G,S8D).

In OCI-AML3, p300i comparatively repressed the expression levels of “inflammation” genes in IC50_r cells, “evolution” genes in IC90_r cells, “stress” genes in short-term BETi-treated cells and those of “degression” genes in general (Fig.6H,S8B,S8E). Conversely, p300i massively boosted “interferon” genes in IC50_r cells only (Fig.6K,S8G). Given that IC50_r cells were most sensitive to p300i, we tested if resistance mechanisms related to upregulation of S100A9, a gene upregulated within the “inflammation” group (Fig.S8E). We either treated cells with the S100A9 inhibitor Paquinimod or created cell lines with inducible RNAi-mediated *S100A9* knockdown. In both settings, this inhibition was significantly higher in IC50_r than IC90_r/DMSO cells (Fig.6I-J,S8F). To test the requirement for the upregulated interferon signature, we induced RNAi-mediated knockdown of the interferon-effector *STAT1* in IC50_r and IC90_r cells before treating them with p300i. Remarkably, p300i failed to inhibit proliferation in *STAT1*-depleted IC50_r cells compared with DMSO-treated controls. Conversely, in *STAT1*-depleted IC90_r cells, p300i significantly suppressed proliferation (Fig.6L,S8H). Taken together, our data demonstrate the temporal and genotypic complexity of p300-induced adaptation following BETi, which evolves continuously and utilizes separate and often transient emergency programs to maintain AML fitness.

### p300 mediates early patterns of resistance to BETi in primary AML samples from a phase I/II clinical trial of Molibresib

**Figure 7:**
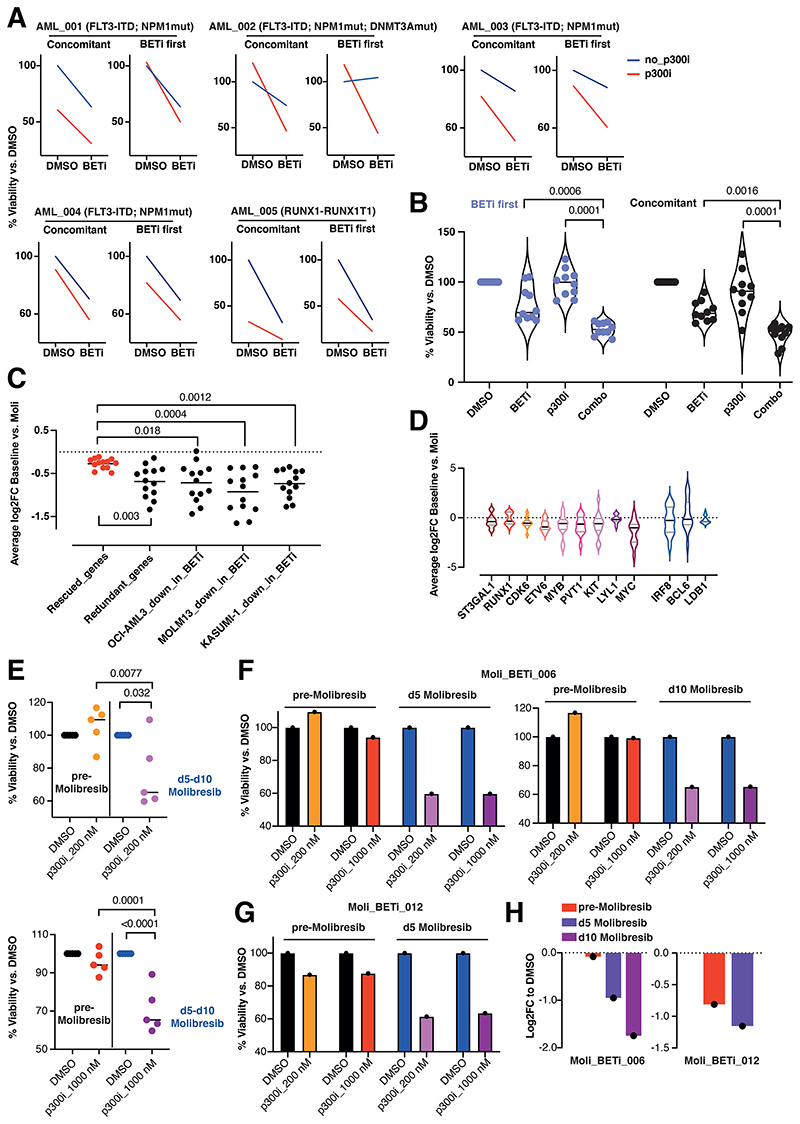
p300 mediates early patterns of resistance to BETi in primary AML samples from a phase I/II clinical trial of Molibresib. A. Individual plots showing treatment efficiency of concomitant or sequential treatment with BETi and p300i in 5 primary AML patient samples. Treatment efficacy was measured with CellTiterGlo^**®**^ at day 4 after commencement of the first treatment. Shown are results from 2 biological replicates for each primary sample. For the sequential treatment mode with BETi first, p300i was added 48hr after treatment commencement. B. Violin plots showing treatment efficacy of concomitant or sequential treatment with BETi and p300i in 5 primary AML patient samples. Shown are results from 2 biological replicates for each primary sample. For the sequential treatment mode with BETi first, p300i was added 48hr after treatment commencement. C. Dot plots of average log2 fold expression changes of the indicated gene sets in 13 patients pre- and early post-dose with Molibresib in a Phase 1/2 clinical trial. Negative values indicate a decrease in expression in the post-dose samples. D. Violin plots of the average log2 fold expression changes of the indicated genes in 13 patients pre- and early post-dose with Molibresib in a Phase 1/2 clinical trial. Negative values indicate a decrease in expression in the post-dose samples. E. Viability after treatment with p300i (either 200 nM - upper panel or 1000 nM -lower panel) or DMSO in primary samples from the Molibresib trial at pre-dose and 5 (for four samples) days or 10 (only for patient Moli_BETi_006, for whom samples were obtained at both day 5 and 10) days post-dose. Shown is the percent viability (measured via CellTiterGlo) to DMSO-treated cells. Due to the limited cell numbers of this clinical trial sample, only one “biological” replicate could be obtained. F.-G. Individual examples of viability after treatment with p300i (200 nM or 1000 nM) or DMSO in the primary samples Moli_BETi_006 (F) or Moli_BETi_12 (G) from the Molibresib trial at pre-dose and 5 days (for both patients) or 10 days (only for patient Moli_BETi_006). Shown is the percentage viability (measured via CellTiterGlo) to DMSO-treated cells. H. Changes in expression of MYC in the same exemplar patient samples Moli_BETi_006 and Moli_BETi_12 at 24 hr of treatment with 200 nM or 1000 nM of p300i. Shown are log2 fold changes to DMSO-treated cells.

In line with results from human cell lines and murine models, we confirmed *ex-vivo* in one *RUNX1-RUNX1T1*-rearranged and 4 *NPM1*-mutated patient samples that sequential BETi followed by p300i treatment significantly inhibited proliferation to a comparable degree in comparison with the concomitant treatment mode (Fig.7A-B).

Additionally, we investigated whether our findings correlated with data from a phase I/II trial testing the BET inhibitor Molibresib as monotherapy in hematologic malignancies, which failed to induce significant clinical responses in AML^18^. Paired RNASeq data at baseline and early post-dose were available for 13 patient samples (Supp.Table_S4). Despite the disparate AML genotypes and their lack of overlap with *RUNX1-RUNX1T1*-rearranged AML, we examined the transcriptional dynamics of our rescued genes (identified from KASUMI-1/SKNO1) following Molibresib dosing. The rescued genes were then compared with the dynamics of other gene-sets within the same patients; a ‘redundant’ gene group consisting of transcripts that consequently decreased upon loss of BRD4 in SKNO1 SLAM-Seq, and the groups that were significantly down-regulated by BETi in KASUMI-1, OCI-AML3 and MOLM-13 as introduced in Figure 3. Remarkably, the decrease of rescued genes appeared blunted in all clinical samples, while all other gene-sets showed significantly deeper downregulation after Molibresib (Fig.7C). A relative preservation of expression was observed for our exemplar genes *ST3GAL1, RUNX1, CDK6, ETV6, MYB, PVT1, KIT, LYL1* and *MYC*, as well as for the OCI-AML3-related putative rescued genes *IRF8, BCL6* and *LDB1* (Fig.7D).

Finally, 4 patient samples taken from the Molibresib trial at day 0 (pre-dose) and matched with day 5 (post-dose) samples were subjected to p300i treatment. In one case, a sample was also available at day 10 post-dose. Compared to the pre-dosed samples, post-dose samples were significantly more sensitive to p300i, correlated with a more profound decrease in *MYC* expression (Fig.7E-H,S9A-C).

Thus, studies in patients treated *in-vivo* and in clinical samples taken from patients exposed to Molibresib corroborate our findings of the efficacy of sequential BET- and p300-inhibition.

## Discussion

After a transient response, the majority of AML patients die from disease relapse^2,50–53^. The emergence of novel clones and the expansion of clones with new mutations—distinct from those at diagnosis—complicate treatment by altering the genetic and epigenetic landscape.

These observations suggest that an understanding of resistance mechanisms is a major unanswered challenge in leukemia research. As resistance mechanisms are likely pleotropic, drug- and genotype-dependent, the importance of this question is amplified by the growing number of promising novel agents^54,55^.

By definition, resistance must initiate under the selective pressure of the induction therapy^56^ and it remains unclear whether acute mechanisms that underpin initial persistence are the same as those that prevail in bulk disease upon re-challenge at relapse. This distinction may have clinical implications: response/survival are far higher for treatment at diagnosis than in relapse and many physicians feel that for some AML there is only “one-shot” at effecting cure. A greater knowledge of acute mechanisms of resistance could therefore allow for the rational development of combinations to prevent relapse^57^. We have addressed these questions, using the evolution of BETi-resistance in sensitive systems.

Our primary objective was to anticipate chronic resistance to BETi by analyzing early adaptation pathways leading to cellular persistence. We identified that p300 rapidly compensates for the loss of transcription at key AML genes, to explain, at least in part, acute persistence. Whilst our experiments focused on p300 to emphasize the translational consequences and to simplify the narrative, we also observed that RUNX1, RUNX1-RUNX1T1 and FLI1, known critical TFs in KASUMI-1 cells^28,30,31,33,35,58^ were enriched at the top rescued sites, in conjunction with p300. These results reconcile with a demonstrated signaling cascade composed of TFs, p300/CREBBP, and BRD4 to support leukemia maintenance, in which TFs and p300/CREBBP recruit/instruct BRD4 to initiate transcription^37^.

p300 activity is redundant with CREBBP at some loci, and their lysine acetyltransferase domains share an almost identical homology^40,59,60^. Therefore, CREBBP may perform a similar function in resistance to BETi. In our study this distinction is somewhat academic as deg_p300 targets both p300 and CREBBP, p300i inhibits the enzymatic function of both p300 and CREBBP and our proposed therapeutic intervention also targets CREBBP-mediated adaptation^40^. Nonetheless, we used the term “p300” solely to ease the narrative, as we do not have evidence whether p300 and CREBBP have common or independent functions as a response to BETi.

In our BETi resistance model, synthetic dependency on p300i varied between models. SKNO1 cells maintained maximum dependency from IC50 to IC90 resistance levels, despite reduced chromatin activity of p300 in IC90-resistant cells. In OCI-AML3, sensitivity to p300i was highest in early resistance stages and gradually declined as full resistance developed. While exploring the mechanisms behind this dynamic resistance and its response to p300i, we noted significant differences in transcriptional responses among AML subtypes. We confirmed the role of NCAM1 in resistance, particularly in *RUNX1-RUNX1T1-*rearranged AML, and identified interferon and inflammatory responses as critical to early resistance in NPM1mut cells, thereby deepening our understanding of transcriptional plasticity in BETi resistance^14,16,22,47,61,62^. Although highly intriguing, several questions remain. Are the inflammatory/interferon signals cell-intrinsic, or do they involve autocrine/paracrine communication effective even in reductionist suspension-based systems? Additionally, it is unclear why certain transcriptional programs are crucial only during early resistance; are they too energy-intensive to sustain, or do they depend on residual BRD4 activity that diminishes over time?

Altogether, we demonstrate that acute resistance to BETi involves a mechanistic feedback to p300, preserving critical leukemia programs. This acute resistance can evolve into fully established resistance where p300 continues to regulate temporal- and genotype-specific programs to maintain leukemic fitness. Additionally, our work establishes a framework for sequential treatment with BETi followed by p300i, a strategy that enhances early synthetic lethality and presents a rational combination designed to prevent relapse.

## Supplementary Material

Supplementary Materials

## Data Availability

RNASeq, ChIPSeq, ATACSeq, SLAMSeq and nucRNASeq data have been deposited in the GEO database under the accession number GSE167163. Anonymized patient data from the NCT01943851 trial were made available by GSK on www.clinicalstudydatarequest.com for research proposals approved by an independent review committee. A data sharing agreement will be required.

## Key points

- A pro-survival transcriptional feedback-loop via p300 enables acute tolerance to BET inhibition.
- p300 also regulates transcriptional networks that lead to chronic resistance to BET inhibition.
- Sequential BET-, followed by p300-inhibition, improves cytotoxicity in AML, and is optimally deployed during earlier stages of resistance-development to BET inhibitors.
