# Supplementary material for: Acute resistance to BET inhibitors remodels compensatory transcriptional programs via p300 co-activation
Source: Blood. Author manuscript; Available in PMC 2025 Dec 23. (PMC7618521; doi:10.1182/blood.2022019306)
Supplement: Supplementary Materials [file EMS211603-supplement-Supplementary_Materials.pdf]

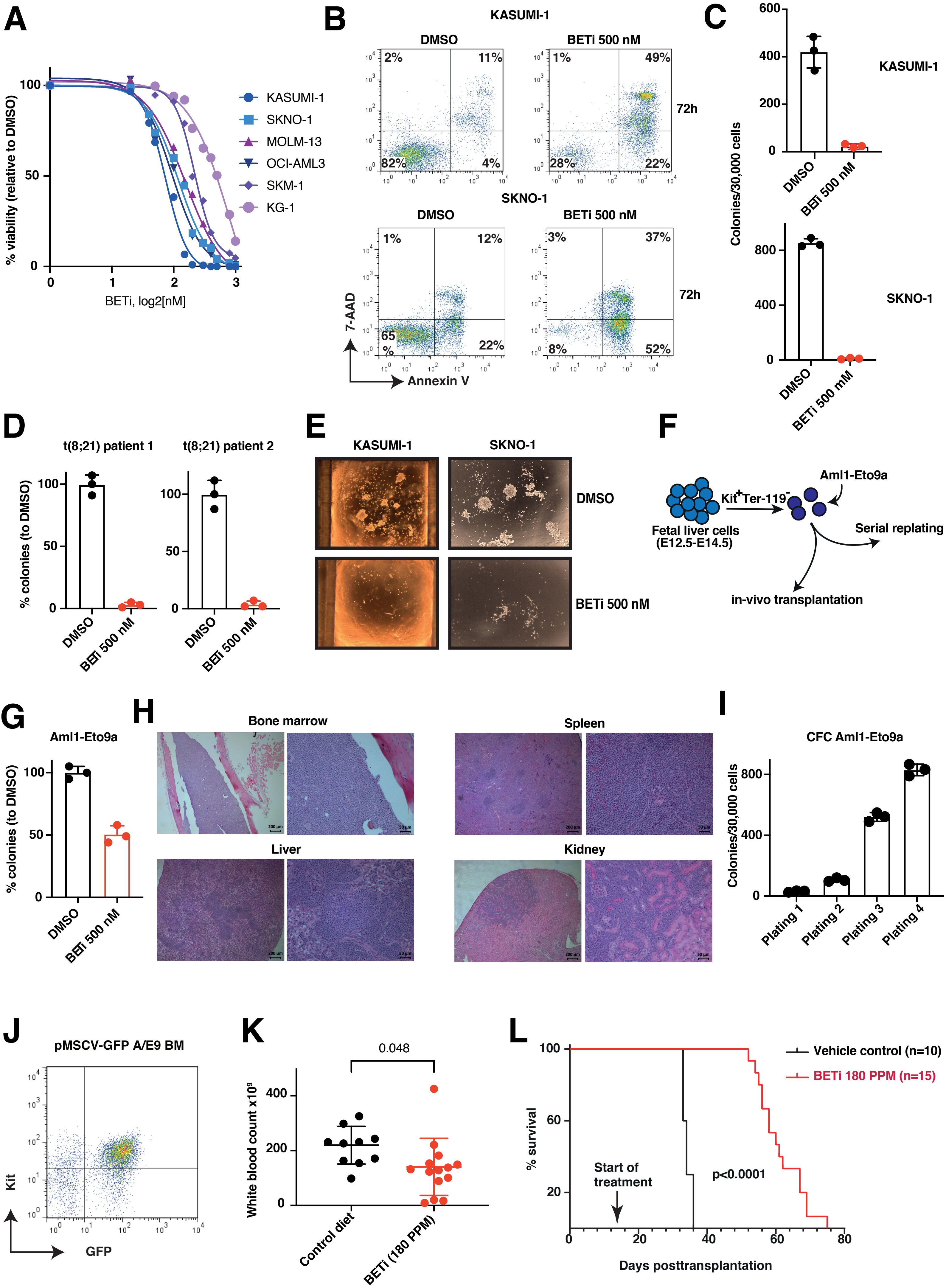

Supplemental Figure 1

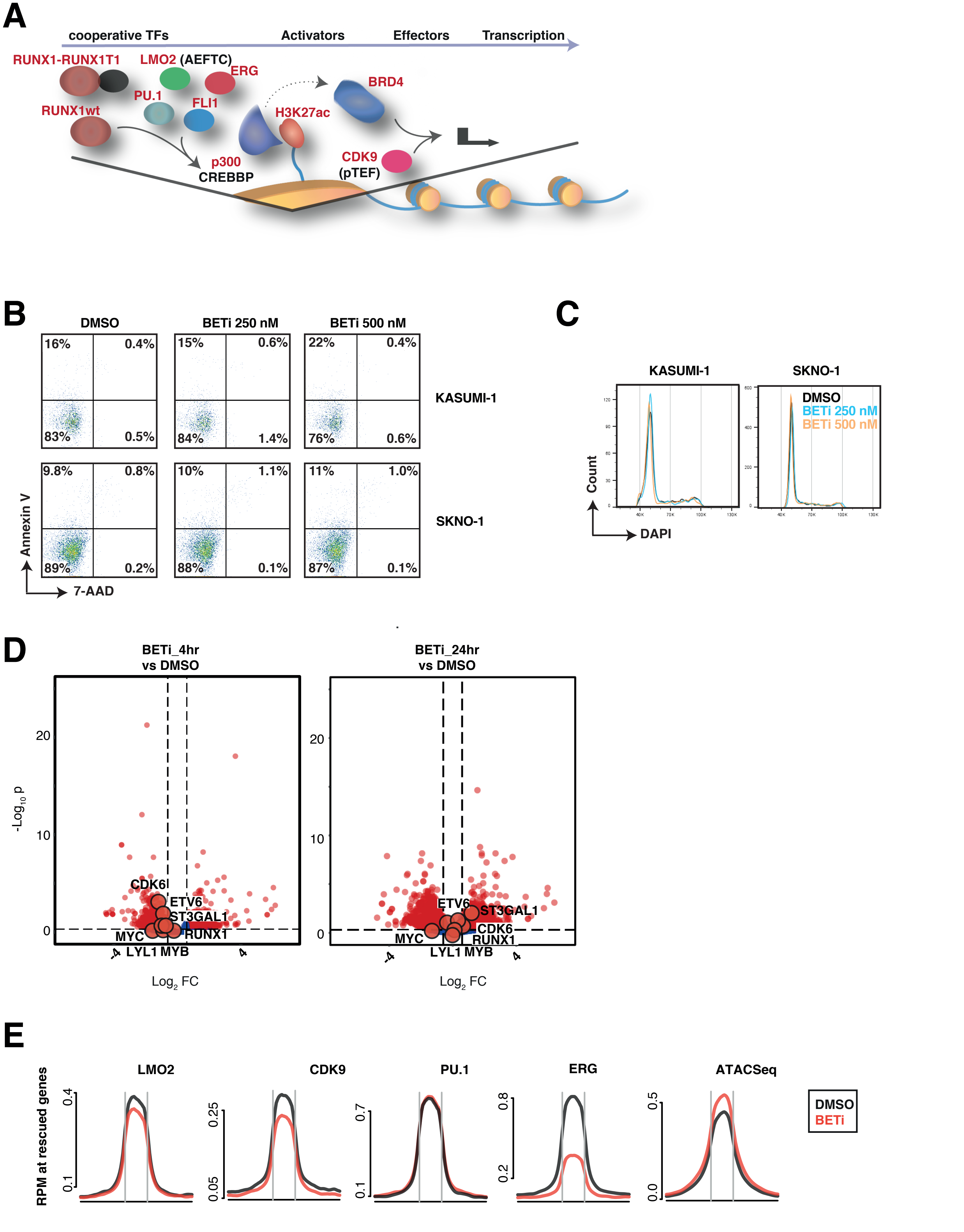

Suplemental Figure S2

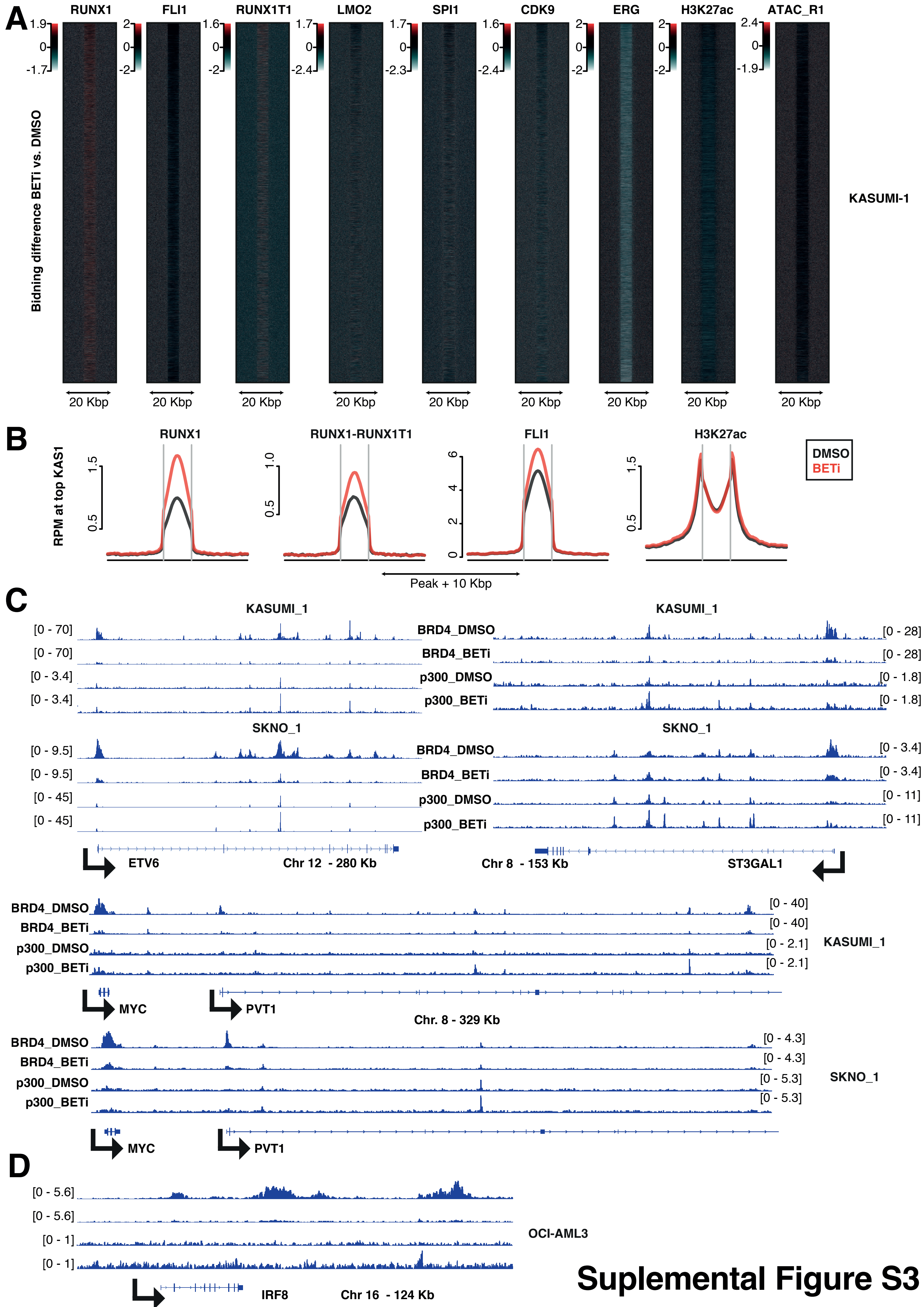

Suplemental Figure S3

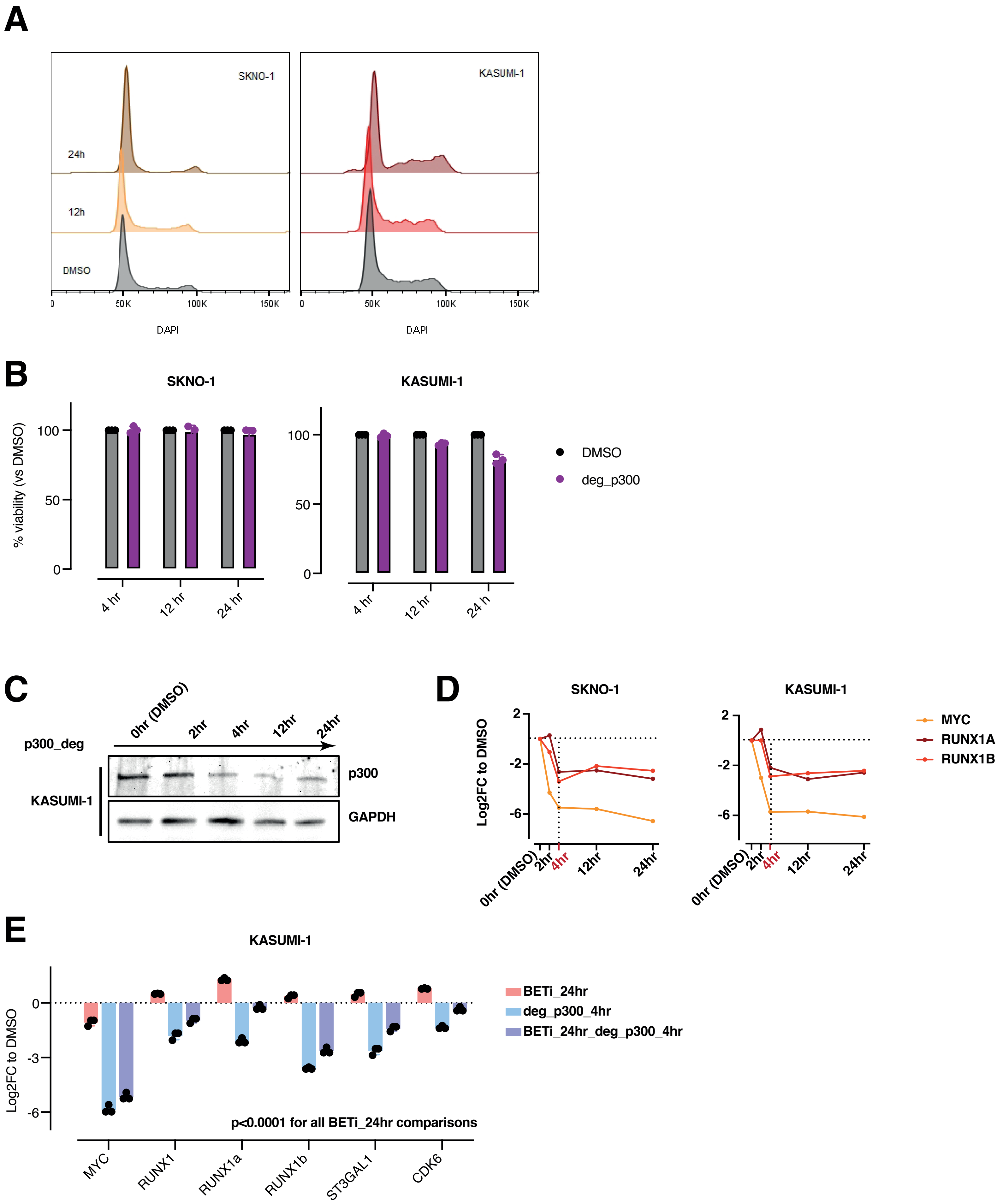

Suplemental Figure S4

A

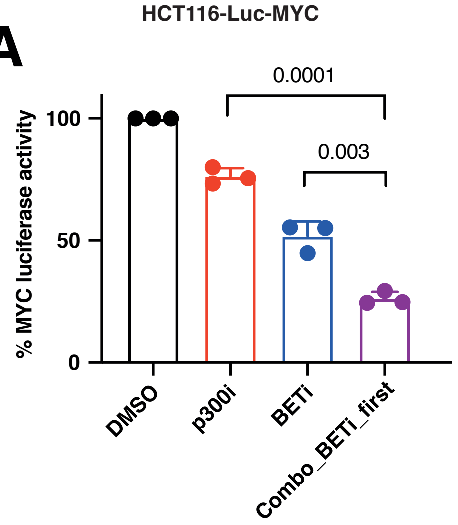

B

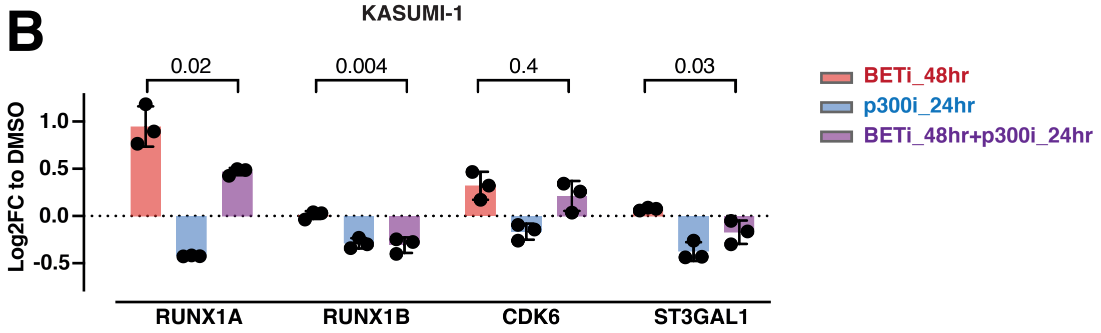

Supplemental Figure S5

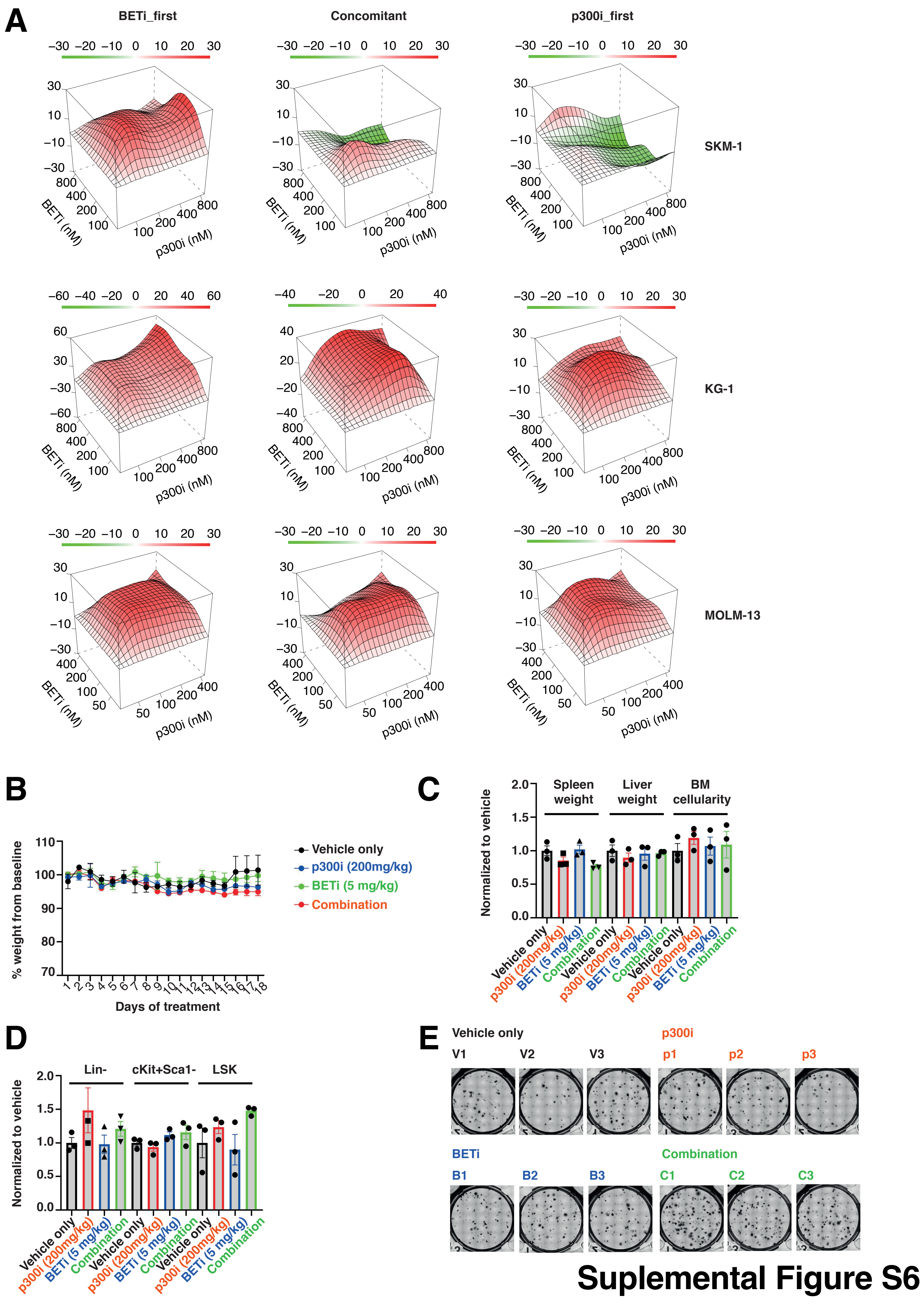

Suplemental Figure S6

A

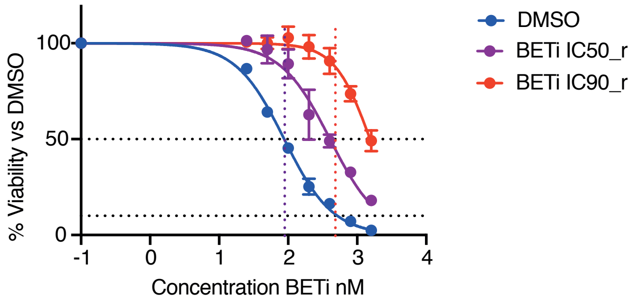

B

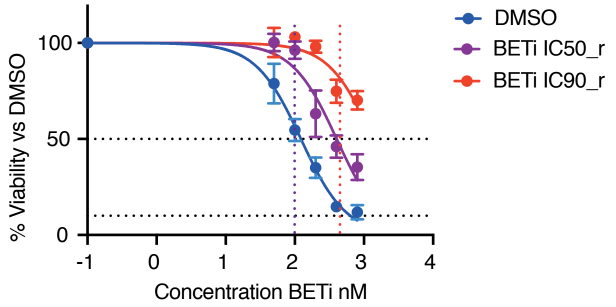

C

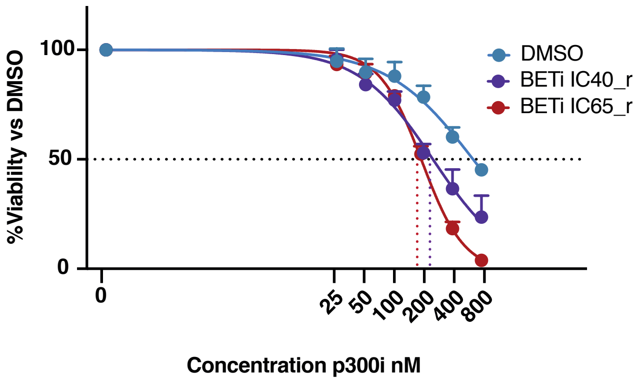

D

	p300i nM
DMSO	616 nM
IC40_r	223 nM
IC50_r	110 nM
IC65_r	182 nM
IC90_r	123 nM

E

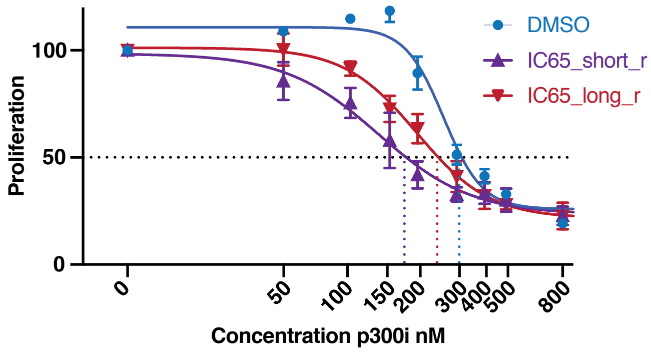

F

	p300i nM
DMSO	304 nM
IC50_r	177 nM
IC65_short_r	173 nM
IC65_long_r	247 nM
IC90_r	275 nM

Supplemental Figure S7

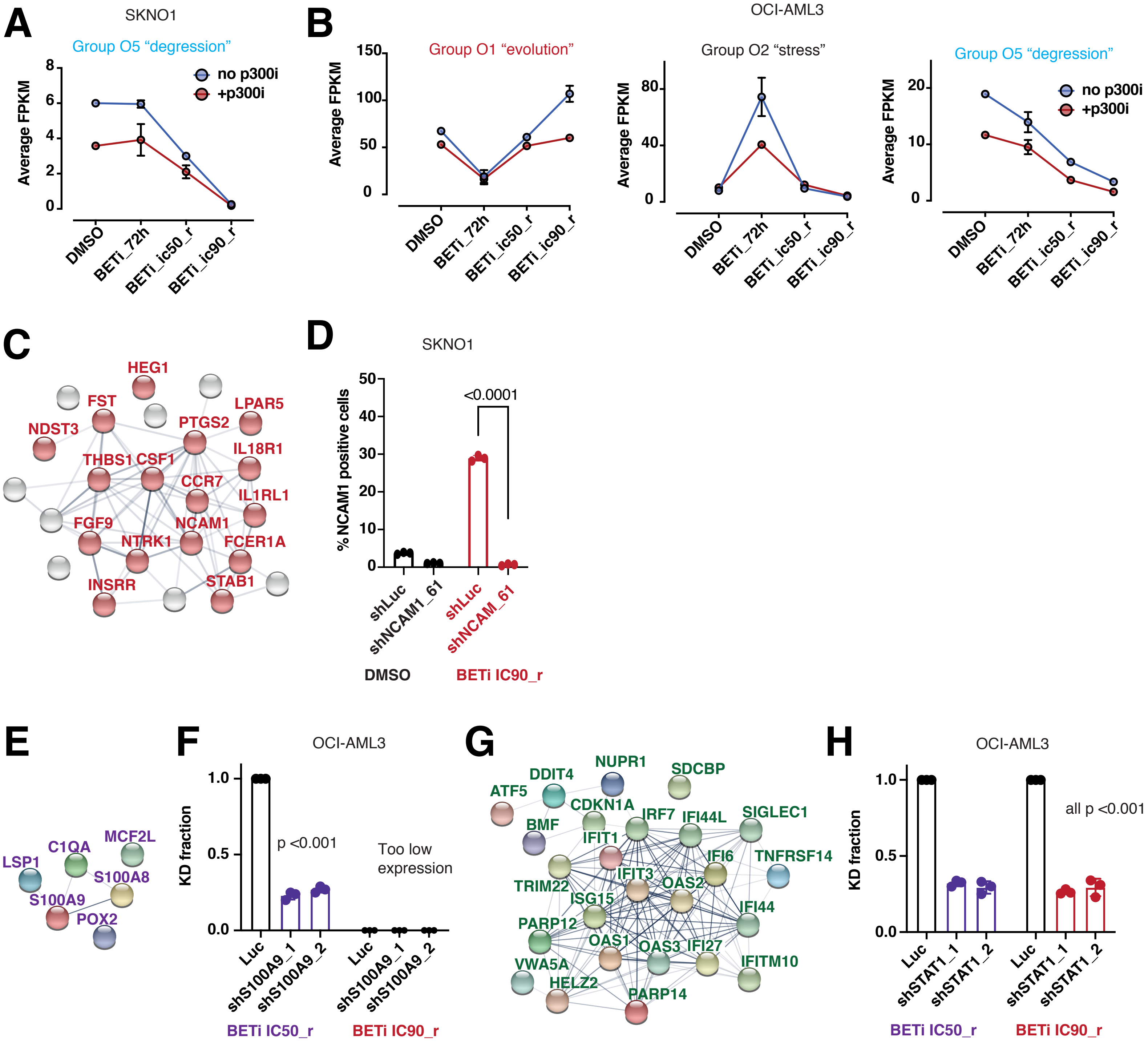

Supplemental Figure S8

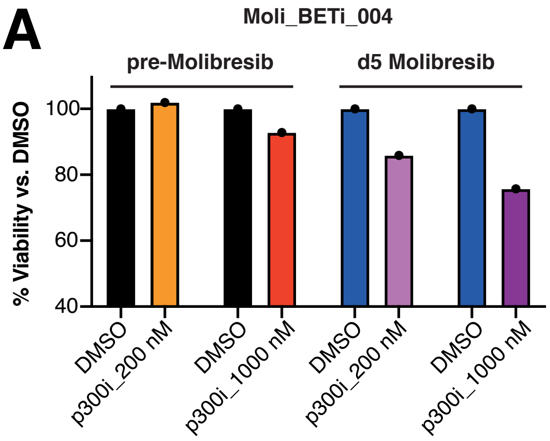

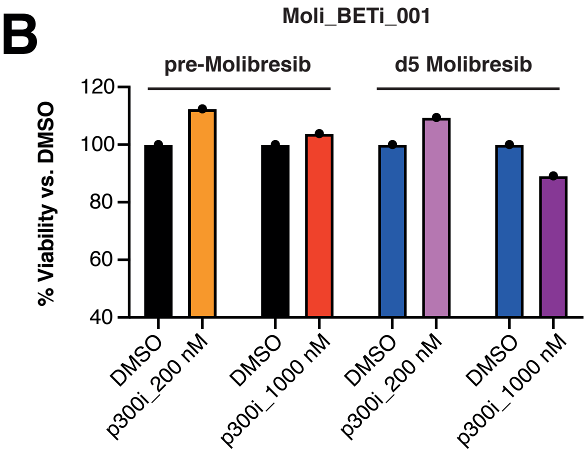

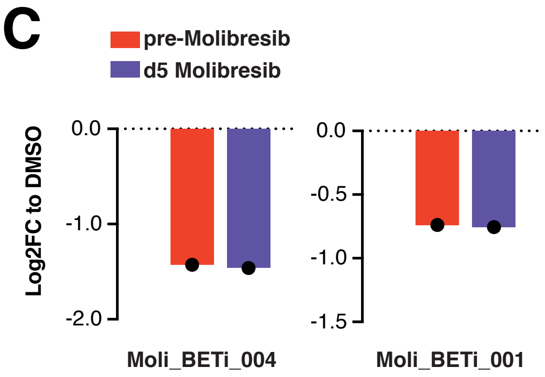

Supplemental Figure S9
